# Viscoelastic Creep of 3D-Printed Polyethylene Terephthalate Glycol Samples

**DOI:** 10.3390/polym17152075

**Published:** 2025-07-29

**Authors:** Leons Stankevics, Olga Bulderberga, Jevgenijs Sevcenko, Roberts Joffe, Andrey Aniskevich

**Affiliations:** 1 Institute for Mechanics of Materials, Faculty of Science and Technology, University of Latvia, Jelgavas Str. 3, LV-1004 Riga, Latvia; olga.bulderberga@lu.lv (O.B.); jevgenijs.sevcenko@lu.lv (J.S.); 2 Division of Materials Science, Luleå University of Technology, S-97187 Luleå, Sweden; roberts.joffe@ltu.se

**Keywords:** 3D printing, PETG, mechanical testing, viscoelastic creep, fused filament fabrication

## Abstract

This article explores the viscoelastic properties of polyethylene terephthalate glycol samples created by fused filament fabrication, emphasising the anisotropy introduced during fabrication. The samples were fabricated with filament direction within samples aligned along the principal axis or perpendicular. A group of samples was loaded with constant stress for 5 h, and a recovery phase with no applied stress was observed. Another group of samples was loaded for 20 h without an additional deformation recovery phase. The continuous constant stress application results on the sample were analysed, and an overall effect of anisotropy on the samples was observed. Several models describing viscoelastic deformation were considered to adhere to experimental data, with the Prony series and general cubic theory models used in the final analysis. The models could describe experimental results up to 50% and 70% of sample strength, respectively. The analysis confirmed the nonlinear behaviour of printed samples under constant stress and the significant effect of anisotropy introduced by the 3D printing process on the material’s elastic properties. The viscoelastic properties in both directions were described using the same parameters.

## 1. Introduction

Fused filament fabrication (FFF) is an exceedingly popular and affordable 3D printing technology [1]. Layer-by-layer material deposition allows for the precise fabrication of complex geometries that would be difficult or impossible to achieve with traditional manufacturing methods, as well as increased speed and cost-effectiveness with which unique objects can be created. FFF is used for many applications, such as building custom gadgets, prototyping before mass production, and creating unique medical prosthetics. Its main advantages are versatility, ease of use, and cost-effectiveness [2]. Some of the most popular printing materials are polylactic acid (PLA), acrylonitrile butadiene styrene (ABS), and polyethylene terephthalate glycol (PETG) [3]. PETG is a commonly used filament due to its high impact resistance, durability, and chemical resistance, making it an excellent candidate for applications requiring mechanical stability.

Although layer-by-layer deposition has advantages over traditional methods, the biggest drawback is the printed sample anisotropy. The 3D-printed parts may display significant directional dependencies in strength, stiffness, and deformation behaviour [4]. A well-known property among 3D printer users is that interlayer adhesion is generally weaker than intralayer [5]. This is the reason it is advised for printed objects to be oriented so that the primary stress axis is located parallel to the printing bed plane, preferably along the printing direction, rather than perpendicularly to the filament direction, where the layered structure of samples is more pronounced. This peculiarity presents a major challenge in engineering applications requiring predictable mechanical performance.

Also, polymers as materials introduce additional challenges in predicting mechanical properties [6] due to their complex internal structure. Materials with long polymer chains tend to deform over time, which can compromise printed object functionality and structural integrity. This deformation can manifest in two primary ways: reversible viscoelastic or irreversible viscoplastic deformation. Slow continuous deformation over extended periods of time under constant stress is called viscoelastic creep [7]. The strain rate during creep decreases, converging on a steady state, with material continuing to deform for a long time. This means that 3D printing might not be suitable for all purposes, especially for objects expected to withstand constant stress over extended periods of time or with very precise tolerances required, such as mechanical gears, structural supports, or mechanical joints. Additionally, material creep can result in material failure at stress levels lower than the yield strength [8].

Heating and cooling during printing can affect the material’s molecular structure, primarily changing material crystallinity [9]. Whether this introduces anisotropy on the molecular level and affects viscoelastic properties, primarily determined by molecular interactions within the polymer chains, is not yet clear. As elastic properties have been proven to be influenced by anisotropy [10], it is important to isolate parameters related to elastic and viscoelastic deformation and research purely the effect 3D printing has on viscoelastic components. Understanding such effects is important for improving the design and durability of 3D-printed components. Studies were made to determine the viscoelastic properties of 3D-printed samples for different materials using different tests and models [11,12,13,14], but the uniqueness of this research lies in the closer investigation of applied models and how these models can handle anisotropy.

The aim of this work is to perform a comparative analysis of various linear and nonlinear viscoelastic creep models, select and apply the most appropriate models to the description of the creep and recovery of unidirectional printed PETG samples, and evaluate the effect of the material anisotropy on the creep.

## 2. Review and Selection of Modelling Approach

**Figure 1:**
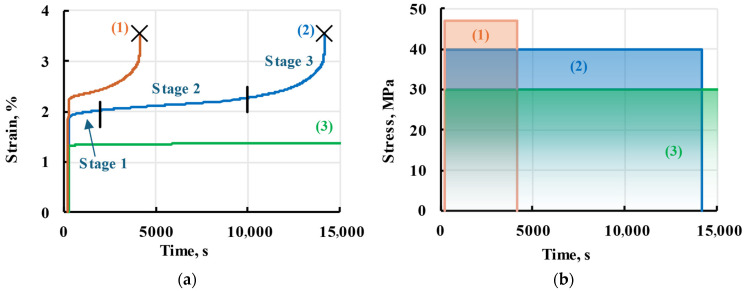
General scheme of a material creep (**a**) under high (1), moderate (2), and low-stress levels (3) and applied stress (**b**). Three creep stages are given for creep at a moderate stress level. Crosses (×) represent samples breaking.

Gradual, time-dependent material deformation when subjected to a constant load over an extended period is called material creep [15]. The creep process is generally divided into three stages, as seen in Figure 1, curve 2 [16,17]. The first stage is a non-uniform creep, as the deformation rate decreases after a high rate during the elastic step. Once the minimal strain rate is achieved, the second stage begins. It has a constant creep rate, and the material deforms proportionally to elapsed time or stays at constant strain, depending on the material and applied stress [18,19,20]. Increased creep rate in the third stage is due to plastic deformations. The material might not follow the general curve if the applied stress is too small, Figure 1, curve 3, or skip over to plastic deformations due to high stress, Figure 1, curve 1. The strain of the first and second creep stages is reversible and is therefore called viscoelastic, which is the focus of this work. A gradual return to original dimensions is present when the applied load is removed, called viscoelastic recovery.

Viscoelastic deformations result from molecule chain deformations, as parts of molecule chains change positions between local energy minimums [6,21]. This phenomenon can be easily observed in amorphous polymers with long molecular chains. When no stress source is applied to a material, molecules can change between states randomly. Still, molecules tend to align themselves with applied stress along the principal stress axis. During the second creep stage, molecules change positions with a constant probability or are at a new equilibrium under low stress. The chains’ reorientation is induced after applying stress to the material sample. Due to thermodynamic processes, the macromolecular chain segments tend to align along the principal stress axis [6,22]. Different chains and configurations affect final strain differently, and it takes different chain sections a different amount of time to deform. Additionally, deformation at any given moment in time is dependent on the applied stress. If deformation is proportional to applied stress, the material exhibits linear viscoelastic properties. Alternatively, the material can exhibit nonlinear viscoelastic properties.

During work different linear and nonlinear models have been evaluated, the results can be seen in later subsections, but for interactive demonstration in Wolfram Mathematica, please view a Supplementary File S1.

### 2.1. Boltzmann–Volterra Theory

A simple way to model the viscoelastic behaviour of a material is to combine Hooke’s law of elasticity.

(1) $$\varepsilon =\frac{\sigma }{E},$$

and Newton’s law of viscosity [19]

(2) $$\dot{\varepsilon }=\frac{\sigma }{3\nu },$$

where σ is the applied stress, ε is the sample strain, *E* is the elasticity modulus, and ν is viscosity. Combining these two equations results in the following:

(3) $$\dot{\varepsilon }=\frac{\dot{\sigma }}{E}+\frac{\sigma }{3\nu },$$

This equation describes a model in which material consists of a series of springs and dashpots; proposed by J. C. Maxwell [23,24]. Suppose dashpots and springs are connected in series. In that case, the model accounts for elastic deformation and subsequent elongation under constant stress and recovery after taking the stress away, but the deformation has no upper boundary. Conversely, a model proposed by Lord William Kelvin and Woldemar Voigt [23,25] imagines a dashpot and a spring connected in parallel and limits the maximum deformation but does not account for elastic deformation at the beginning. A more complex hybrid model was created to account for edge cases but did not give precise values observed in the experiments. The solutions to equations derived from these models were in the form of the Volterra function, which led Ludwig Boltzmann to create a general solution to the viscoelastic model called the Boltzmann–Volterra theory:

(4) $$\varepsilon (t)=\frac{\sigma (t)}{E}+\int_{0}^{t}K(t-\tau )\sigma (\tau )d\tau ,$$

where *K*(*t*) is the creep function. The expression *K*(*t*) is described by different functions referred to as viscoelastic kernels representing different models [26].

The choice of the viscoelastic kernel is heavily based on the investigated material, as well as on computational power and required precision [20,27].

### 2.2. Linear Viscoelastic Kernels

There are three types of linear kernels: kernels based on power law, exponential kernels, and fractional exponential kernels. Some of the most popular viscoelastic kernels were used in the research. Representative curves with arbitrarily chosen kernels’ parameters are shown to visualise possible creep and recovery curves typical to each model. For these representative curves, applied stress was modelled as a step function, where constant stress was applied at time $t_{0}$ = 0 and taken away after $t_{1}$ = 10 arbitrary units (a.u.), where *t* is normalised time. Stress and elasticity modulus, unless stated otherwise, are equal to 1.

Model parameters α, β, *a*, *c*, and *n* are constants of several kernels but have different physical meanings and values for different models.

#### 2.2.1. Power Law Kernels

**Figure 2:**
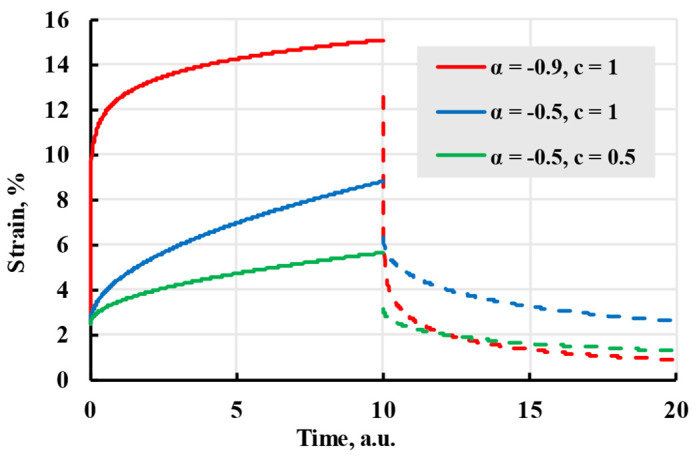
The creep (solid lines) and recovery process (dashed lines) are modelled with the Duffing kernel with coefficients given in the legend.

Based on the shape of the creep function during stage 1 and the beginning of stage 2, it is reasonable to assume that the power law can adequately describe the function [28]:

(5) $$K(t-\tau )=a{\left(t-\tau \right)}^{n},$$

where a and n are constants. This assumption would break down for longer timeframes, so Boltzmann then proceeded to create his kernel:

(6) $$K(t-\tau )=\frac{c}{t-\tau },$$

where c is constant. However, it is not defined when the variable of integration τ is equal to time *t*, necessitating further modifications. A set of kernels was further developed. The most successful of which was the Duffing kernel [26], which takes the form

(7) $$K(t)=ct^{\alpha },-1<\alpha <0,$$

where c and α are constants. As seen in Figure 2, constant α changes the steepness of curves at the beginning of the creep, while constant c determines the strain rate at a longer elapsed time. Integrating Boltzmann–Volterra Equation (4) with momentary stress results in a deformation equation:

(8) $$\varepsilon (t)=\frac{\sigma }{E}+\frac{\sigma c}{1+\alpha }\left({\left(t-t_{1}\right)}^{\left(1+\alpha \right)}-{\left(t-t_{0}\right)}^{\left(1+\alpha \right)}\right).$$

The model created in 1918 by Georg Duffing [29] describes deformation at stages 1 and 2 well enough but has the same problem as the Maxwell model—it does not have an upper deformation boundary. Therefore, it cannot describe deformations of samples where stage 2 creep remains constant.

#### 2.2.2. Exponential Kernels

**Figure 3:**
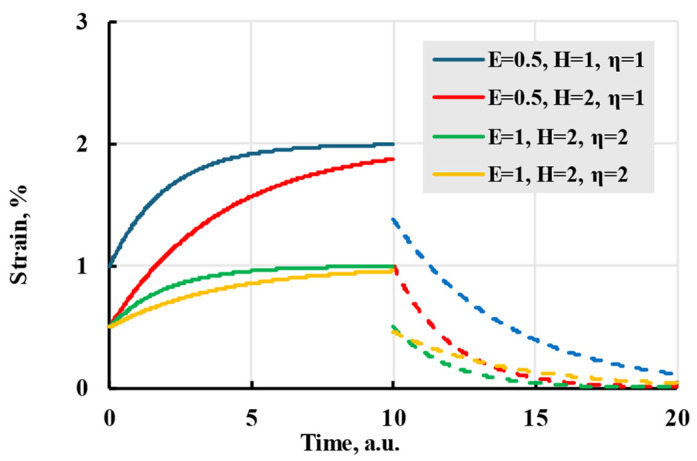
The creep (solid lines) and recovery process (dashed lines) are modelled with an exponential kernel with coefficients given in the legend.

Another way to evaluate deformation is to use the exponential law instead of the power law. A simple exponential kernel can be written as

(9) $$K(t-\tau )=\frac{H-E}{\eta H^{2}}e^{-\frac{E(t-\tau )}{\eta H}},$$

where H is the elastic modulus at a very large time and η is relaxation time. The effect each parameter has on the modelled strain can be seen in Figure 3. The resulting analytical solution for deformation is

(10) $$\varepsilon (t)=\frac{\sigma }{E}+\sigma \frac{\left(H-E\right)}{EH}\left(e^{-\frac{E(t-t_{1})}{\eta H}}-e^{-\frac{E(t-t_{0})}{\eta H}}\right).$$

Though this kernel can easily model material creep at low stress, the model has difficulties describing deformations for proportionally changing creep stage 2, Figure 1. A solution for this problem is to use Boltzmann’s superposition principle: deformation due to creep of the material is based on loading history, and the total creep is the sum of the creep deformation caused by each loading [30]. By making a sum of exponential kernels, it is possible to recreate a linear creep of stage 2, while having control over deformation at stage 1 [31,32]. The kernel form is analogous to the Prony analysis method; therefore, the resulting kernel is called the Prony series:

(11) $$K(t-\tau )=\sum_{i=1}^{N}\frac{A_{i}}{E\eta_{i}}e^{-\frac{t-\tau }{\eta_{i}}},$$

where $\eta_{i}$ is relaxation time and $A_{i}$ is the amplitude of the respective relaxation time. Together, $A_{i}$ and $\eta_{i}$ form the relaxation spectrum of the material. As a result, the analytic formula, derived from (4) using this viscoelastic kernel, is

(12) $$\varepsilon (t)=\frac{\sigma (t)}{E}+\int_{0}^{t}\sigma (\tau )\sum_{i=1}^{N}\frac{A_{i}}{E\eta_{i}}e^{-\frac{t-\tau }{\eta_{i}}}d\tau .$$

**Figure 4:**
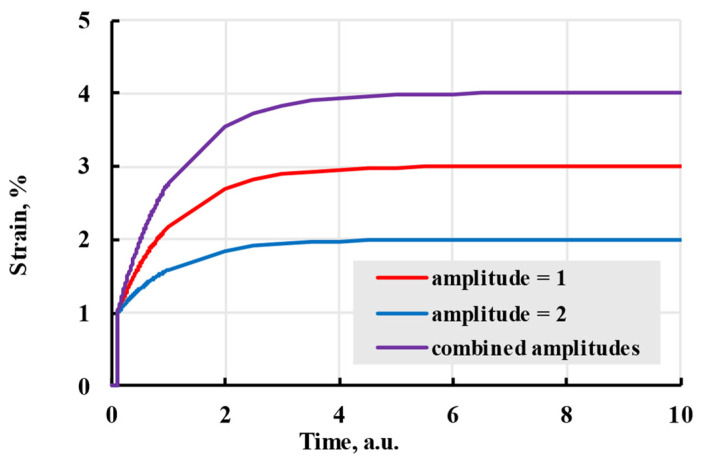
The viscoelastic creep is modelled with a Prony kernel with different amplitudes.

Viewing the formula apart from the physical interpretation, relaxation time amplitudes can be viewed as deformation added after time on the scale of relaxation time. Adding new terms with the same relaxation time to the analytical formula adds that amplitude to the deformation during creep (Figure 4).

**Figure 5:**
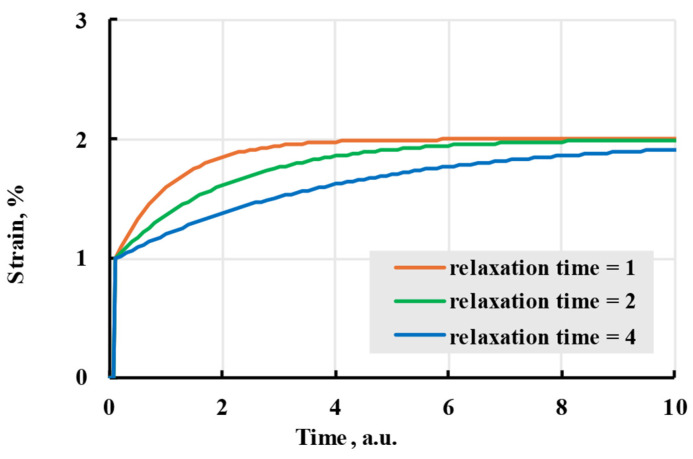
The viscoelastic creep is modelled with a Prony kernel with relaxation times of 1, 2, and 4 arbitrary units.

**Figure 6:**
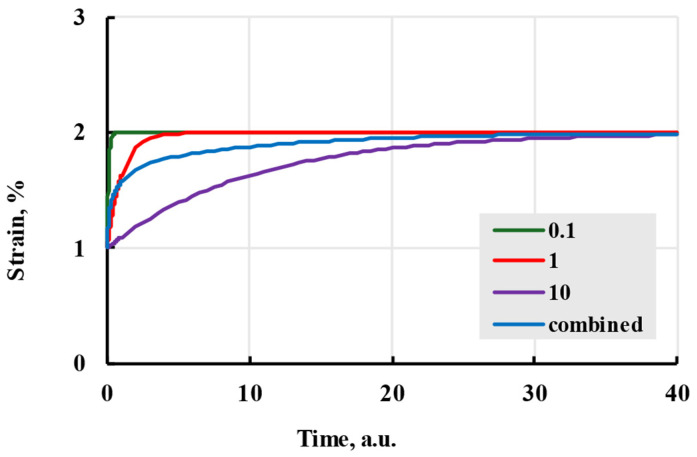
The viscoelastic creep is modelled with a Prony kernel; comparison with relaxation times in arbitrary units being powers of 10 given in the legend.

Different relaxation times indirectly represent the thermodynamic movement of different molecular chain parts. Larger relaxation times relate to slower polymer conformation changes and slower deformation speed. If all amplitudes of terms are the same, all terms will reach the same deformation in time. The difference in speed can be minimal, even when doubling the relaxation time (Figure 5). To limit possible options, relaxation times are usually chosen as powers of 10 or evenly spaced through the experiment time [33]. The advantages of using the Prony series kernel can be seen when observing the curves given by each term separately and their combination with total additional deformation equal to amplitudes of separate terms. Figure 6 shows that combination gives a deformation speed higher than the term with the biggest relaxation time but lower than the smallest.

**Figure 7:**
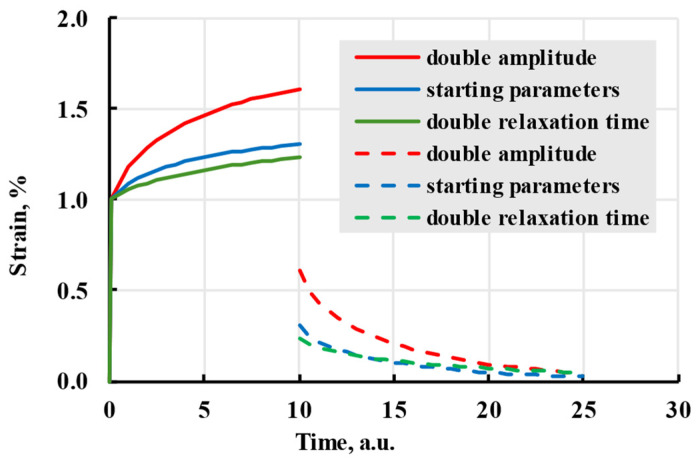
The creep (solid lines) and recovery process (dashed lines) are modelled with the Prony kernel with coefficients given in the legend.

Although only material creep was discussed to illustrate the effects different parameters have on the model, material recovery is also affected by the changes in kernel parameters (Figure 7). The optimal size of the relaxation spectrum is dependent on the time scale, but generally, more terms result in better prediction [14].

#### 2.2.3. Fractional Exponential Kernels

As one of the most important properties of the viscoelastic model is the ability to predict deformation based on stress history, fractional calculus was applied to find a unique solution for the deformation problem [34,35]. Using fractional calculus, kernels that do not fully adhere to exponential or power law can be calculated. These kernels are called fractional or fractional-exponential kernels. One of the easier-to-implement fractional kernels is the Rzhanitsin kernel, created in 1968 [36]:

(13) $$K(t-\tau )=ce^{-\beta (t-\tau )}{\left(t-\tau \right)}^{\alpha }.$$

The model

(14) $$\varepsilon (t)=\frac{\sigma }{E}+\sigma \frac{c}{\beta }\left(\frac{{\left(t-t_{1}\right)}^{\alpha }{\left(\beta \cdot (t-t_{1})\right)}^{-\alpha }}{\Gamma (\alpha +1,\beta \cdot (t-t_{1}))}-\frac{{\left(t-t_{0}\right)}^{\alpha }{\left(\beta \cdot (t-t_{0})\right)}^{-\alpha }}{\Gamma (\alpha +1,\beta \cdot (t-t_{0}))}\right).$$

**Figure 8:**
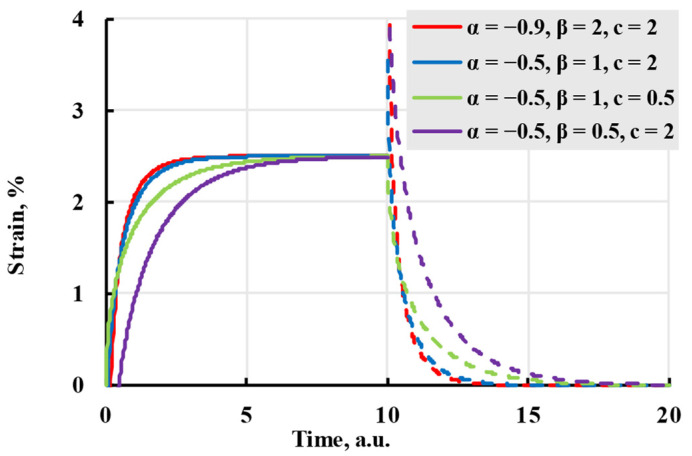
The creep (solid lines) and recovery process (dashed lines) are modelled with the Rzhanitsin kernel with coefficients given in the legend.

Although it described the viscoelastic creep better, it has difficulty modelling creep recovery from viscoelastic deformation and is more complex to calculate. The difficulties of the model can be seen in Figure 8, where chosen parameters, although they follow the general form of viscoelastic creep, do not give a result substantially different from the elastic model, as well as the curve using parameters α = −0.5, β = 0.5, *c* = 2, has a strain starting at negative values and recovery starting higher than the end of creep.

Yu. N. Rabotnov proposed the most overarching kernel in 1948 [37]:

(15) $$K(t-\tau )={\left(t-\tau \right)}^{\alpha }\sum_{n=0}^{\infty }\frac{\beta^{n}{\left(t-\tau \right)}^{n(1+\alpha )}}{\Gamma [(n+1)(1+\alpha )]},-1<\alpha <0.$$

**Figure 9:**
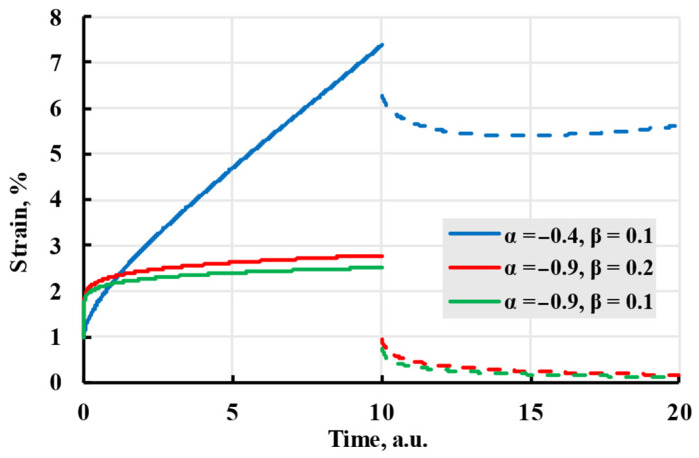
The creep (solid lines) and recovery process (dashed lines) are modelled with the Rabotnov kernel with coefficients given in the legend.

The advantage of this kernel is that it can produce a wide number of different kernels depending on the values of α and β. Although sources claim [26] that the result can be summed up to infinity and constant α can take any value from −1 to 0, attempts at modelling during this research suggested that the sum does not converge and has an upper limit of summands, as well as the value of α is dependent on β, as seen in Figure 9. The curve “α = −0.4, β = 0.1” strains graph curves during recovery and, after reaching a minimum, tends to grow, which is contrary to expected behaviour.

One of the last overviewed kernels was the Abel kernel, which uses the Abelian integral:

(16) $$K(t-\tau )={\left(t-\tau \right)}^{\alpha }\frac{{\left(t-\tau \right)}^{\alpha }}{\Gamma [1+\alpha ]},-1<\alpha <0.$$

**Figure 10:**
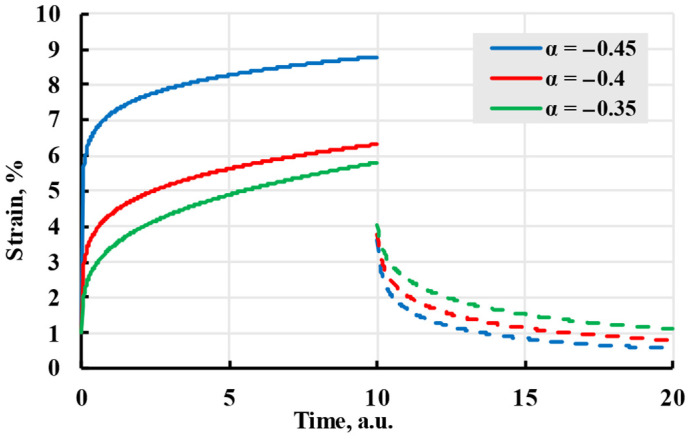
The creep (solid lines) and recovery process (dashed lines) are modelled with the Abel kernel with coefficients given in the legend.

As seen in Figure 10, a model calculated using the Abel kernel creates curves that resemble a general form of viscoelastic creep.

#### 2.2.4. Linear Kernel Comparison

For the purposes of this research, the kernel that could describe strain for stages 1 and 2, including situations where it becomes constant, is required. Therefore, a kernel that has high adherence to the exponent is preferable. Additionally, a kernel that can be easily computed and does not have a large number of parameters, as well as an elastic modulus defined after elastic deformation, is preferable for ease of use. In Table 1, the better-fitting kernels are coloured in green, the worse-fitting in red, and some intermediate results are yellow. It was evaluated that the Prony series would be best for this work.

### 2.3. Loading Rate Effect on Creep Process

**Figure 11:**
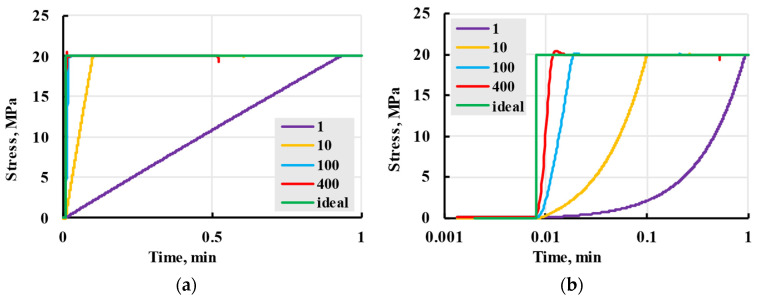
The loading rate problem in creep tests is illustrated in the example of PLA White tests at stress 20 MPa applied on ZWICK testing machine in stress–time axes (**a**) and semi-logarithmic stress–time axes (**b**). Numbers in the legend are loading rates in mm/min.

Some real-life problems may occur during testing, one of the most prominent being non-instantaneously applied stress during sample loading, as seen in Figure 11. For example, samples of 20 MPa stress loading at different speeds were observed on a ZWICK 2.5 testing machine (ZwickRoell GmbH & Co. KG, Ulm, Germany). At a high loading rate, a testing machine overshoots the target stress level, causing problems in determining elastic strain and the beginning stage of creep [38]. This overshoot is seen for a loading rate of 400 mm/min (red line); it is less pronounced for a loading rate of 100 mm/min (blue line) compared with ideal stepwise loading (green line). The loading stage in Figure 11 intentionally started at time *t*_0_ = 0.008 min and shifted from the zero point to be clearly visible in semi-logarithmic coordinates.

This requires using a lower loading rate, but if the sample is loaded too slowly, viscoelastic creep, which happens during the loading stage, has to be taken into account [39]. The overshoot disappears for loading rates of 10 and 1 mm/min.

Lower loading rates need more time (up to 1 min), which delays the ideal creep process. All lines are delayed from the “Ideal” (green) line in Figure 11. Some viscoelastic strain could also be observed during this period. For deformation to be considered purely elastic, a ratio of loading time t and relaxation time τ must be lower than 1: $\frac{t}{\tau }<1$ [40]. This relation also dictates that models can not include relaxation times lower than the loading time unless creep is accounted for during loading. To investigate the effect of possible relaxation times lower than loading time on the overall model, a case when the stress is assumed to be a linear time function (linear loading) was considered:

(17) $$\sigma (t)=\left\{\begin{array}{cc} gt+h, & 0<t\leq t_{1}\\ const, & t_{1}<t \end{array}\right.,$$

where *g* and *h* are the linear function coefficients, *t*_1_ is the time when the nominal creep stress was achieved and after that moment, the stress is unchanged. It was accepted for modelling that the loading stage started at *t*_0_ = 0.

After adding the values of stress (17) to (4), it can be divided into two summands: before reaching $t=t_{1}$ and after that,

(18) $$\varepsilon (t)=\frac{gt+h}{E}+\sum_{i=1}^{k}\frac{A_{i}}{\eta_{i}E}\int_{0}^{t}\left(g\tau +h\right)e^{-\frac{t-\tau }{\eta_{i}}}d\tau ,\;0\leq t<t_{1},$$

(19) $$\varepsilon (t)=\frac{\sigma }{E}+\sum_{i=1}^{k}\frac{A_{i}}{\eta_{i}E}\left(\int_{0}^{t_{1}}\left(g\tau +h\right)e^{-\frac{t-\tau }{\eta_{i}}}d\tau +\int_{t_{1}}^{t}\sigma e^{-\frac{t-\tau }{\eta_{i}}}d\tau \right),\;t_{1}\leq t.$$

Integrating both (18) and (19) results in

(20) $$\varepsilon (t)=\frac{gt+h}{E}+\sum_{i=1}^{k}\frac{A_{i}}{E}\left(g\left(\eta_{i}\left(e^{-\frac{t}{\eta_{i}}}-1\right)+t\right)-ce^{-\frac{t}{\eta_{i}}}+h\right),\;0\leq t<t_{1},$$

(21) $$\varepsilon (t)=\frac{\sigma }{E}+\sum_{i=1}^{k}\frac{A_{i}}{E}\left(e^{-\frac{t}{\eta_{i}}}\left(e^{\frac{t_{1}}{\eta_{i}}}\left(g\left(t_{1}-\eta_{i}\right)+h\right)+g\eta_{i}-h\right)+\sigma \left(1-e^{-\frac{t-t_{1}}{\eta_{i}}}\right)\right),\;t_{1}\leq t.$$

**Figure 12:**
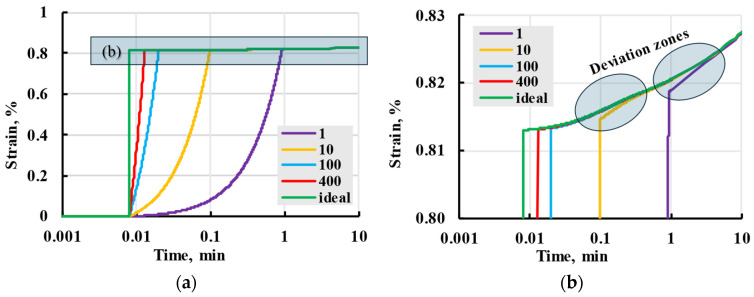
Modelled viscoelastic strain (**a**) for instantaneous ideal stepwise loading (green line) and various loading rates (numbers in the legend in mm/min) and zoomed-in curves (**b**).

The deformation that appears during the loading stage in explicit form is expressed by (20) and inexplicitly included in (21). The linear loading model was compared with stress loading as the Heaviside function (instantaneous loading). For the relaxation spectrum with the lowest relaxation time of 0.1 min, deformation curves seen in Figure 12 were calculated. The differences in deformation at the creep stage when the loading step is finished can be observed for different loading speeds. For high loading speeds of 400 and 100 mm/min, the deformation is indistinguishable from the instantaneous ideal loading. The red and blue lines coincide with the green line for the creep stage. For low loading speeds of 10 and 1 mm/min, the deviation is noticeable. Still, the lines converge with the deformation of instantaneous loading within a period of the next nearest power of ten, i.e., 1 min for 10 mm/min and 10 min for 1 mm/min (the deviation zones are shown).

Based on the results of this modelling, it could be concluded that the effect of the loading rate on the overall creep process can be ignored beyond one order of time after the first relaxation time and is presumed to be a part of elastic deformation. In other words, it could be neglected for tests performed during this research.

### 2.4. Stepwise Loading

While the loading scheme (17) is enough for simple cases, the general multistep loading is more useful and easier to scale up for different tests. For this purpose, a general form of stepwise loading (22) was considered.

(22) $$\sigma \left(t\right)=\left\{\begin{array}{ll} \sigma_{1} & t_{1}\leq t<t_{2}\\ \sigma_{2} & t_{2}\leq t<t_{3}\\ \ldots & \\ \sigma_{N-1} & t_{N-1}\leq t<t_{N}\\ \sigma_{N} & t_{N}\leq t \end{array}\right..$$

Calculating integral (12) with loading (22), as seen in Appendix A, leads to an analytical solution:

(23) $$\varepsilon \left(t\right)=\frac{\sigma_{N}}{E}+\overset{k}{\underset{i=1}{\sum }}\frac{A_{i}}{E}\left[\overset{N-1}{\underset{n=1}{\sum }}\sigma_{n}e^{-\frac{t}{\eta_{i}}}\left(e^{\frac{t_{n+1}}{\eta_{i}}}-e^{\frac{t_{n}}{\eta_{i}}}\right)+\sigma_{N}\left(1-e^{\frac{t_{N}-t}{\eta_{i}}}\right)\right].$$

**Figure 13:**
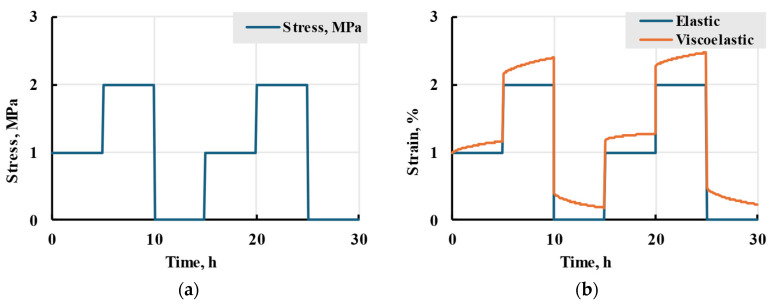
An applied example of a loading scheme (24) (**a**) and corresponding strain comparison for elastic (1) and viscoelastic models (23) (**b**).

As an example of a loading programme, a six-step stress application is considered, as seen in Figure 13a. In this case, the stepwise function with time in hours takes the form:

(24) $$\sigma \left(\tau \right)=\left\{\begin{array}{ll} \sigma_{1}=1 & 0\leq t<5\\ \sigma_{2}=2 & 5\leq t<10\\ \sigma_{3}=0 & 10\leq t<15\\ \sigma_{4}=1 & 15\leq t<20\\ \begin{array}{l} \sigma_{5}=2\\ \sigma_{6}=0 \end{array} & \begin{array}{l} 20\leq t<25\\ 25\leq t \end{array} \end{array}\right.,$$

where stress is in MPa. The loading starts at *t* = 0 h in a given case. For an example loading programme (24), Equation (23) will result in a calculated viscoelastic strain seen in Figure 13b, where elastic strain is also given for comparison.

The obtained equation can be easily used for creep modelling with different stepwise loading histories, including creep-recovery cases. Applying stepwise loading combined with stress–time superposition allows for predicting the creep process for times well over what was available for the experiments [41]. Still, it is beyond the scope of this research.

It has to be mentioned that for some polymer materials, the elastic modulus is also a stress-dependent function, i.e., E=E(σ). For this purpose, a model with step-dependant elastic modulus was implemented:

(25) $$\varepsilon \left(t\right)=\frac{\sigma_{N}}{E_{N}}+\overset{k}{\underset{i=1}{\sum }}A_{i}\left[\overset{N-1}{\underset{n=1}{\sum }}\frac{\sigma_{n}}{E_{n}}e^{-\frac{t}{\eta_{i}}}\left(e^{\frac{t_{n+1}}{\eta_{i}}}-e^{\frac{t_{n}}{\eta_{i}}}\right)+\frac{\sigma_{N}}{E_{N}}\left(1-e^{\frac{t_{N}-t}{\eta_{i}}}\right)\right].$$

### 2.5. Nonlinear Models

Depending on the material, applied stress, and temperature, the material exhibits nonlinear viscoelastic properties [26], instead of the linear discussed in the previous section. Nonlinear viscoelastic models help to model additional deformation due to microcracks, plasticisation due to moisture absorption, and change in elastic properties of materials approaching the glass transition temperature. Nonlinear models also allow us to describe irreversible deformations of stage 3 of linearly viscoelastic materials.

#### 2.5.1. Power Law Model

Multiple approaches were made to create nonlinear viscoelastic models. One of the easier-to-implement approaches is to use a power law, similar to how it was conducted in the linear approach (5). The difference is, instead of being a kernel within the Volterra formula, nonlinear power law, also known as Findley’s power law (1976), describes the whole deformation as a power function [20,42]:

(26) $$\varepsilon (t)=\varepsilon_{0}+\varepsilon_{1}t^{n},$$

where $\varepsilon_{0}$ and $\varepsilon_{1}$ are stress-dependent creep constants, $\varepsilon_{0}$ is elastic deformation of a material, n is stress-independent creep constant, that is generally less than 1. Nadai and Prandtl and later Findley suggested incorporating nonlinearity explicitly into the model so $\varepsilon_{0}$ and $\varepsilon_{1}$ can be rewritten using as

(27) $$\varepsilon_{0}=m\sinh\left(\frac{\sigma }{\sigma_{0}}\right),$$

(28) $$\varepsilon_{1}=k\sinh\left(\frac{\sigma }{\sigma_{1}}\right),$$

where $m,\;\sigma_{0},\;k,\;\sigma_{1}$ are stress-independent material constants at a constant temperature [43]. Substituting (27) and (28) into (26) results in

(29) $$\varepsilon (t)=m\sinh\left(\frac{\sigma }{\sigma_{0}}\right)+kt^{n}\sinh\left(\frac{\sigma }{\sigma_{1}}\right).$$

**Figure 14:**
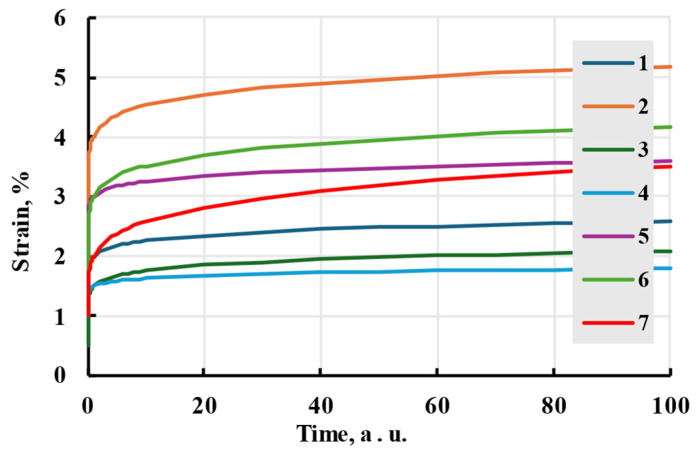
The creep process is modelled with the Findley nonlinear viscoelastic model with different stress-independent constant values. The values of constants can be seen in Table 2.

**Figure 15:**
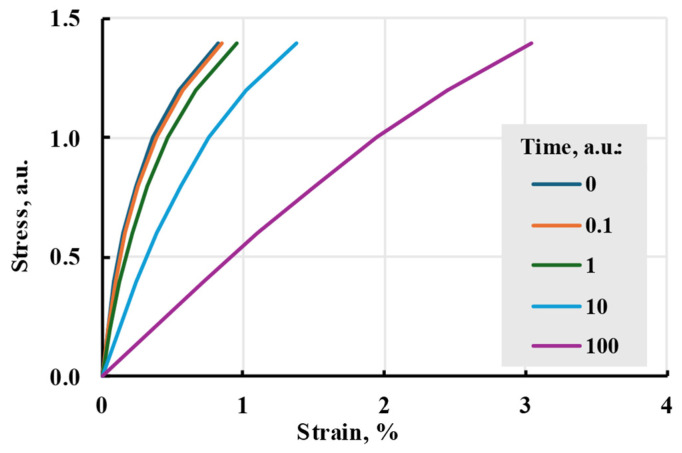
Stress–strain dependences (creep isochrones) at given times.

The power law is rather imprecise and only gives a general form of nonlinear viscoelastic behaviour. The effect of stress-independent constants on the model can be seen in Figure 14. The nonlinearity of the model is shown in Figure 15.

**Table 2:** Coefficient values are used for constructing strain curves in Figure 14.

Curve Number	σ, Pa	σ_0_, Pa	σ_1_, Pa	*m*	$k,\;s^{\frac{1}{n}}$	*n*
1	1	10	10	0.1	0.1	0.1
2	2	10	10	0.1	0.1	0.1
3	1	20	10	0.1	0.1	0.1
4	1	10	20	0.1	0.1	0.1
5	1	10	10	0.2	0.1	0.1
6	1	10	10	0.1	0.2	0.1
7	1	10	10	0.1	0.1	0.2

#### 2.5.2. Relaxation Function Models

Another approach is to assume behaviour to be an integral of the relaxation function, proposed by Leaderman, H. (1943) [44]:

(30) $$\varepsilon (t)=\int K(t-\tau )\frac{d}{dt}\Psi \left(\sigma_{t}\right)d\tau ,$$

where $\Psi \left(\sigma_{t}\right)$ is a stress-dependant function. The general form was later applied to different models, including Rabotnov, Breuller, and Schapery. The R. Schapery model (1964) is one of the most common models of nonlinear viscoelastic behaviour [45]:

(31) $$\varepsilon (t)=g_{0}\frac{\sigma (t)}{E_{0}}+g_{1}\int_{0}^{t}\Delta K(\Phi -\Phi^{\prime })\frac{d(g_{2}\sigma (\tau ))}{d\tau }d\tau ,$$

where $g_{0},\;g_{1},\;g_{2}$ are nonlinear stress [20], temperature, and moisture [46] dependent material constants, Φ and $\Phi^{\prime }$ are shifted stress-dependent time scales:

(32) $$\begin{array}{l} \Phi (t,\sigma )=\int_{0}^{t}\frac{dt}{a_{\sigma }(t)}\\ \Phi^{\prime }(t,\sigma )=\int_{0}^{t}\frac{d\tau }{a_{\sigma }(\tau )} \end{array},$$

where $a_{\sigma }$ is a stress-specific time shift factor. The general form of Boltzmann–Volterra theory can be recovered by substituting nonlinear parameters with 1: $g_{0}=g_{1}=g_{2}=a_{\sigma }=1$ and ΔK(Ψ)=K(t). The nonlinearity is mainly introduced through parameters $g_{2}$ and $a_{\sigma }$, as they govern stress-dependent changes. Although comprehensive, the Schapery model was not modelled during this research, as, for more accurate prediction, it would require a closer inspection of environmental factors [46,47], which was not performed.

When $g_{0}=g_{1}=g_{2}=1$, but $a_{\sigma }\neq 1$, the time–stress superposition principle is obtained. In the simplest case, time is linearly dependent on stress, resulting in general Boltzmann–Volterra to take the form:

(33) $$\varepsilon (t)=\frac{\sigma (t)}{E}+\int_{0}^{t}K(a_{\sigma }(t-\tau ))\sigma (\tau )d\tau ,$$

**Figure 16:**
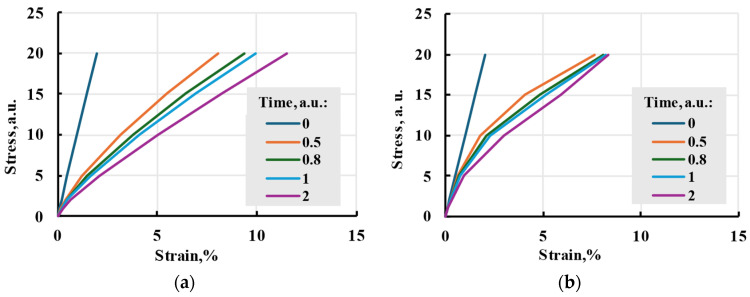
Time–stress analogy model isochrones with linear time shift coefficient dependence on stress $a_{\sigma }=1.5\sigma +1.5$ (**a**) and base 10 exponential $a_{\sigma }=10^{0.1\sigma }$ (**b**).

For the model to be nonlinear, $a_{\sigma }$ is not a constant. During modelling, attempts were made to give $a_{\sigma }$ linear and exponential dependence on stress. No specific function for time correction coefficient dependency on stress was found. So, attempts were made to model nonlinear behaviour using the Prony series and linear and base 10 exponential (Figure 16) dependencies of the time shift coefficient on stress. The resulting creep curve is similar to the linear model, but modelled relaxation processes happen faster, mimicking skipping steps 1 and 2 in Figure 1.

#### 2.5.3. Main Cubic Theory Model

Another approach is to use the Boltzmann–Volterra theory and assume that nonlinearity stems from loading history having an effect and changing the properties of new loading steps [6]. The first assumption is that two loading steps are connected, but it can be extrapolated to include the effects of three or more loading steps. In general form, for impulses at appropriate time moments $\tau_{j}$, viscoelastic kernel *K_j_* of stress impulses can be written as follows [48,49,50]:

(34) $$\begin{array}{l} \varepsilon (t)=\frac{\sigma }{E}+c_{1}\int_{0}^{t}\sigma (\tau )\;K_{1}(t-\tau )d\tau +c_{2}\int_{0}^{t}\int_{0}^{t}\sigma (\tau_{1})\;\sigma (\tau_{2})K_{2}(t-\tau_{1},t-\tau_{2})d\tau_{1}d\tau_{2}+\\ c_{3}\int_{0}^{t}\int_{0}^{t}\int_{0}^{t}\sigma (\tau_{1})\;\sigma (\tau_{2})\sigma (\tau_{3})K_{3}(t-\tau_{1},t-\tau_{2},t-\tau_{3})d\tau_{1}d\tau_{2}d\tau_{3}+\ldots \end{array},$$

where *c_j_* are material constants.

When observed in the same time moments (*s*_1_ = *s*_2_ = …*s*_n_), (40) takes the form:

(35) $$\begin{array}{l} \varepsilon (t)=\frac{\sigma }{E}+c_{1}\int_{0}^{t}\sigma (\tau )\;K_{1}(t-\tau )d\tau +c_{2}\int_{0}^{t}\sigma^{2}(\tau )K_{2}(t-\tau )d\tau +c_{3}\int_{0}^{t}\sigma^{3}(\tau )K_{3}(t-\tau )d\tau +\ldots \end{array}.$$

Only the terms containing stresses in odd powers are kept in for materials with the same deformational properties in tension and compression (35). In the simplest case, it can be assumed that the viscoelastic kernels are equal. Then, for *σ* = const, the time-dependent function is

(36) $$K(t-\tau )=K_{1}(t-\tau )=K_{3}(t-\tau )=\ldots .$$

A number of terms in (34) determine the degree of nonlinearity. In the simplest case, the deformation is calculated using linear and cubic terms in the formula:

(37) $$\varepsilon (t)=\frac{\sigma (t)}{E}+\int_{0}^{t}(c_{1}\sigma (\tau )+c_{3}\sigma^{3}(\tau ))K(t-\tau )d\tau .$$

The parameters of the model are determined from the results of creep tests at different stresses [51,52]. In this research, the Prony series kernel was used, similar to the linear viscoelastic case:

(38) $$\varepsilon (t)=\frac{\sigma (t)}{E}+\int_{0}^{t}(c_{1}\sigma (\tau )+c_{3}\sigma^{3}(\tau ))\sum_{i=1}^{N}\frac{A_{i}}{E\eta_{i}}e^{-\frac{t-\tau }{\eta_{i}}}d\tau .$$

**Figure 17:**
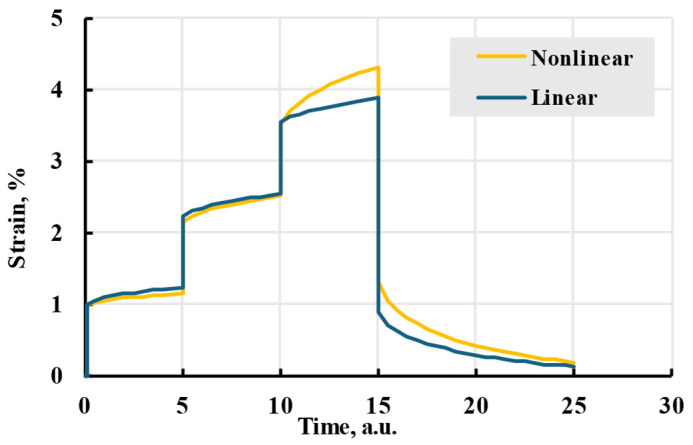
Nonlinear cubic and linear viscoelastic models with the same relaxation spectrum and applied stress.

**Figure 18:**
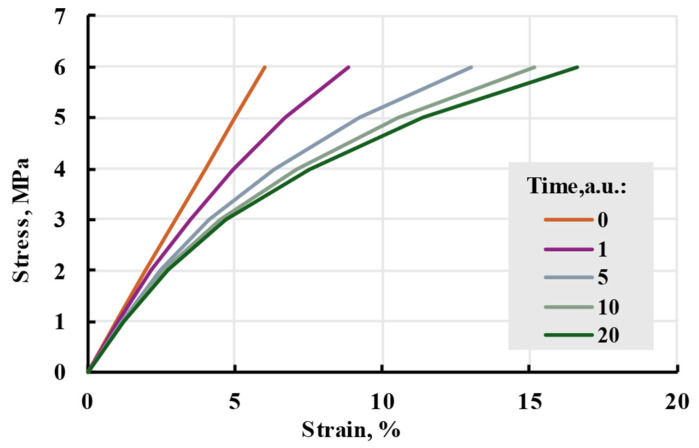
Isochrones of the main cubic model at different times.

In this research, the Prony series kernel was used in the model. It can be compared to the linear model seen in Figure 17. The model shows smaller strains at low stress but a bigger one at higher stress. The nonlinearity of the model can be seen in isochrones in Figure 18.

#### 2.5.4. Nonlinear Model Comparison

Similarly to linear models, an evaluation of nonlinear models was made, as seen in Table 3. Among the evaluated models, stress–time analogy and main cubic theory had the more desirable parameters. The ability to compare results to linear models, medium computational complexity and only two nonlinear parameters made the main cubic theory the best model for this work.

## 3. Materials and Methods

### 3.1. Samples

**Figure 19:**
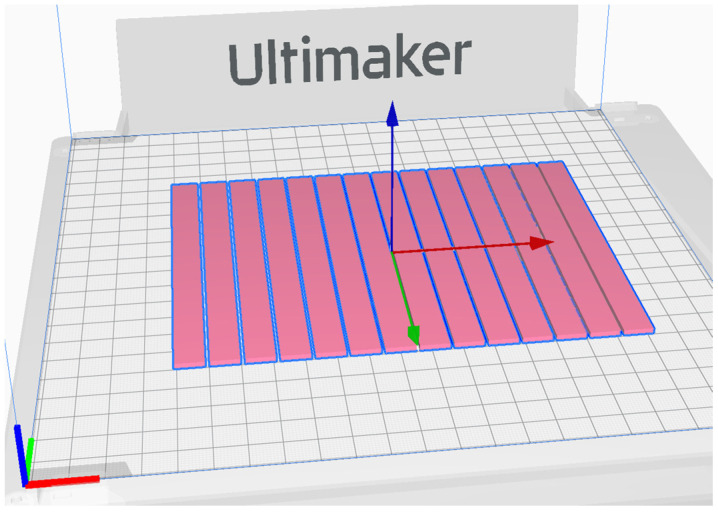
Screenshot of Y samples in slicing programme.

**Figure 20:**
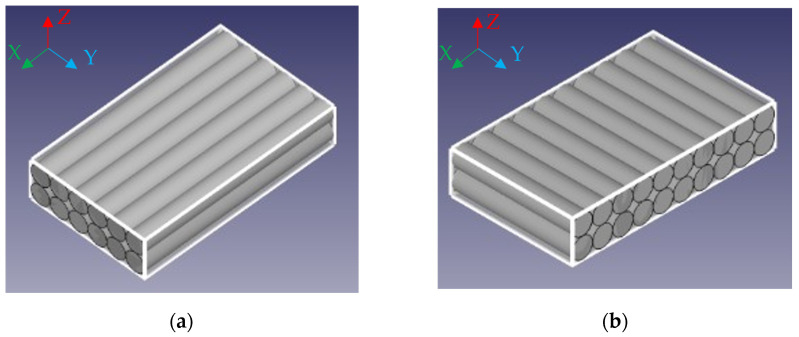
Model of filament alignment in X (**a**) and Y (**b**) samples.

The 2.85 mm diameter PETG filament produced by Devil Design [53], was used for sample printing using Ultimaker S5 printer (Ultimaker B.V., Ultrecht, The Netherlands) with a 0.4 mm diameter nozzle. Samples were printed on a glass bed with an adhesive spray 3DLAC (Laboratorios 3D Print, S.L., Zamora, Spain) coating for better first layer adhesion and to prevent warping. Sample 3D models were sliced using Ultimaker Cura 4.9.1 software. The nozzle temperature was set to 235 °C and the bed temperature to 85 °C. The print head speed was 20 mm/s. The layer thickness was set at 0.1 mm. The infill pattern was set to “Lines”, fibre width (“Line width”) was set to 0.35 mm, and infill density was set to 100%. All layers were printed in the same direction and on the same printing path (Figure 19). Shell and Top/Bottom thickness settings were set to zero. These printer settings provided the unidirectional orientation of all filament fibre in all specimens and uniform distribution within any specimen’s cross-section. Two groups of rectangular bar-shaped specimens having a length of 120 mm and a cross-section size of 3 × 10 mm were printed with filament orientation along the sample principal axis (X) and the principal axis (Y) (Figure 20).

Previous research investigated the effect of 3D printing introduced anisotropy on the elastic properties of the material [10]. Samples X have an elastic modulus of 2.33 ± 0.02 GPa and a strength of 50.44 ± 0.09 MPa. Samples Y have an elastic modulus of 2.19 ± 0.08 GPa and a strength of 22.48 ± 4.61 MPa.

### 3.2. Creep Test

All samples were tested using the Zwick 2.5 universal testing machine according to the standards ASTM D6992-16 [54] and ASTM D2990-01 [55]. The loading speed of samples was 100 mm/min. A gauge zone of 25 mm and a starting grip-to-grip separation of 75 mm. The samples were kept in a dry environment, and silica gel desiccant ensured low relative humidity. Average temperature during the tests was 21.3 ± 0.3 °C. Average humidity during the tests was 39 ± 1%. The discussion about temperature and humidity was not included in the article, as a larger focus at this stage of research was on the creep modelling without consideration of environmental factors, but representative graphs of temperature and humidity during experiments can be viewed in Supplementary Materials Figures S1 and S2.

The measurement error of an extensometer was ±1 µm (0.001%), and the 2.5 kN load cell had an error of less than 1%. This results in strain and stress errors that are barely readable on a graph.

Short-time creep tests of 5 h duration were made with an additional 15 h of recovery. It was assumed that a recovery phase three times longer than the loading phase was sufficient for material to recover after the creep, as no significant strain changes were observed after 15 h, although it should be noted that in creep tests with multiple loaded stages, a recovery stage four times larger than the loaded stage was advised, similar to the time required for evaluation of viscoplastic properties of the material [21,55]. In total, 16 X-direction and 12 Y-direction samples were tested.

Samples used for long-term tests were subjected to constant stress for 20 h, after which creep recovery was not controlled. These tests were performed to further evaluate the accuracy of modelling based on short-term tests. Six X-direction and three Y-direction samples were tested. More tests were performed with X samples, as more stress levels were applied to X samples overall. Applied stress levels and their relation to the strength of the samples can be seen in Table 4.

## 4. Results and Discussion

### 4.1. Elastic Response of Stress Loading and Unloading

**Figure 21:**
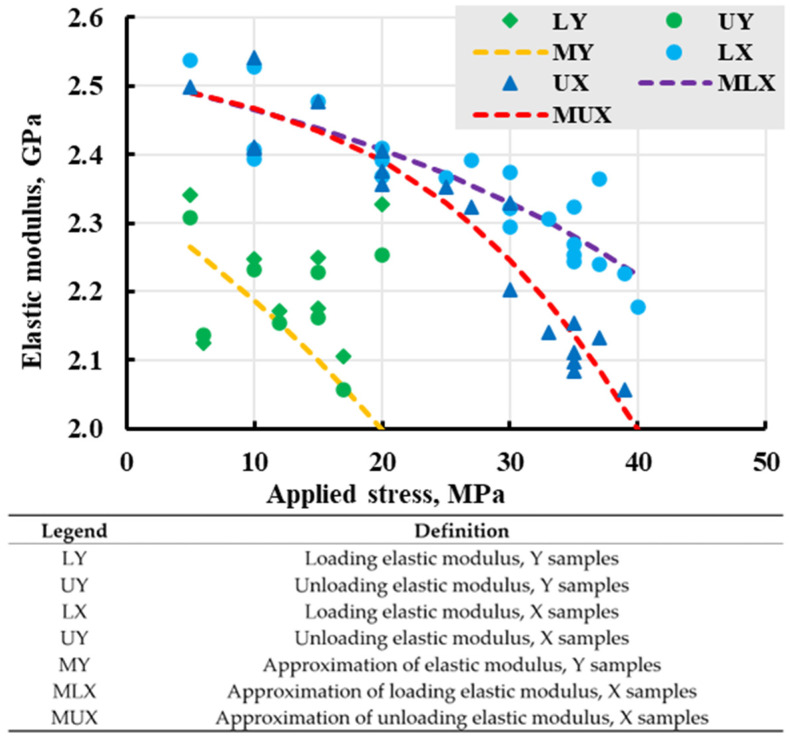
Change in elastic moduli vs. applied stress with approximated best fit for every series. Legend is explained in a table.

One of the indicators of nonlinear polymers’ behaviour is the change in the elastic response during stress loading and unloading. In essence, this is a secant elastic modulus equal to the ratio of conventionally instant elastic strain to applied stress. Several assumptions have been made in this case. One of them is the effect of loading rate on creep, which was discussed earlier. For linear elastic models, responses should be equal, in contrast with nonlinear models [56]. The response can be expressed as a “loading elastic modulus”, which is calculated as a relation between deformation at the end of the loading step and applied constant stress, and “unloading elastic modulus” as a relation between the strain difference from the end of a stress holding step to the moment the machine detects “zero” stress applied to the sample and removed constant stress. After plotting the resulting elastic moduli in Figure 21, it was observed that the responses for the loading and unloading steps were different. The elastic moduli for both responses also decreased with applied stress. The effect of anisotropy can be observed with absolute values of quasi-elastic modulus, but the rate of change stays similar based on similar values of trendlines. The best-fitting approximations for each orientation (Figure 21) were considered when modelling the viscoelastic properties of samples.

In Figure 21, all blue dots and triangles belong to X-direction specimens, and green dots and triangles belong to Y-direction. Blue and green dots indicate elastic modulus during the loading stage and triangles during the unloading stage. Each pair of dot–triangle belongs to one specimen. Visible scatter of the data results from the whole chain of manufacturing and testing.

The values of the elastic modulus of the loading and unloading stages are close for low and moderate stresses. In contrast, the unloading modulus is noticeably less than for that loading stage for high stresses. This difference between the loading and unloading elastic moduli at higher applied stress can be attributed to the accumulation of residual strain, which will be discussed in the next section.

### 4.2. Residual Strain

**Figure 22:**
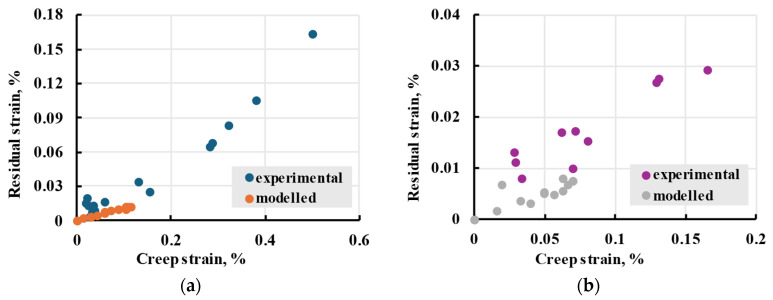
The residual strain against the creep strain of X (**a**) and Y (**b**) samples after 5 h creep and 15 h recovery.

After sample creep recovery during short-term tests, some residual strain was left. This residual strain consisted of non-recovered viscoelastic (insufficient time) and plastic deformation, including internal sample damage and material ageing during creep [57]. To evaluate the overall residual strain, the strain at the end of the recovery was plotted against the creep strain—the full strain at the end of the creep step, with subtracted elastic deformation (Figure 22). A steady increase in residual strain with increased creep strain can be observed. Calculated results using the linear model were plotted next to experimental data to evaluate the amount of plastic to leftover viscoelastic strain.

The results show how additional irreversible deformation occurred within samples subjected to higher strain. They show that plastic deformations need to be accounted for after creep reaches 0.1% for X samples and 0.05% for Y. Based on experimental data, 0.1% creep was observed on samples put under stress more than 50% of the sample’s strength. Similar behaviour was observed during viscoelastic experiments of other materials [58], which indicated a material hardening after the first application of stress.

### 4.3. Viscoelastic Creep Modelling

The obtained data show that in 5 h for both the X and Y samples, under applied stress of 50% of the sample strength, the creep strain is equal to roughly 5% of the sample elastic strain, or 1/20 part. In 20 h, this strain had doubled. At a stress of 70% of the sample strength at 5 h, creep strain in X samples reached 25% of the sample elastic strain or 1/4 part, but for Y samples reached only 7%. The strain increased to 30% for X and 8% for Y.

#### 4.3.1. Linear Model

**Figure 23:**
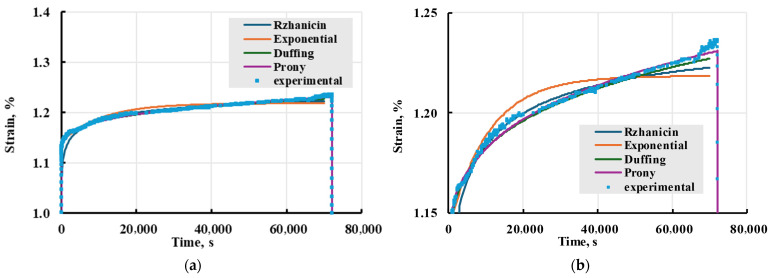
Comparison of viscoelastic models with different kernels given in the legend with experimental data (**a**) and zoomed-in curves (**b**). Creep test for representative PETG X sample loaded to 33 MPa.

Different models were used to describe the experimental strain, as mentioned in Section 2.2.4 and in Table 1. To determine kernels that better adhere to experimental results, modelled curves were plotted next to PETG viscoelastic test result data for representative X sample loaded to 33 MPa in Figure 23. The evaluation showed that Prony and Duffing kernels best describe the experimental results. Combined with what is shown in Table 1, Prony series is the best kernel for this work.

**Figure 24:**
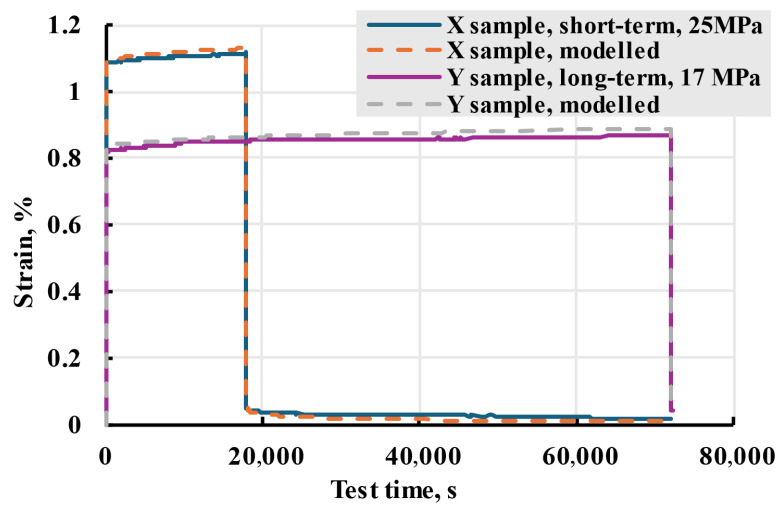
Representative long-term and short-term X and Y creep tests (solid lines) and modelled strain (dashed lines).

Using the Boltzmann–Volterra theory and the Prony series, the deformation on the printed PETG samples was modelled. Modelling was conducted in MS Excel Visual Basic. Representative results of experimental data and modelling can be seen in Figure 24. The model adequately describes the creep process for both cases.

**Figure 25:**
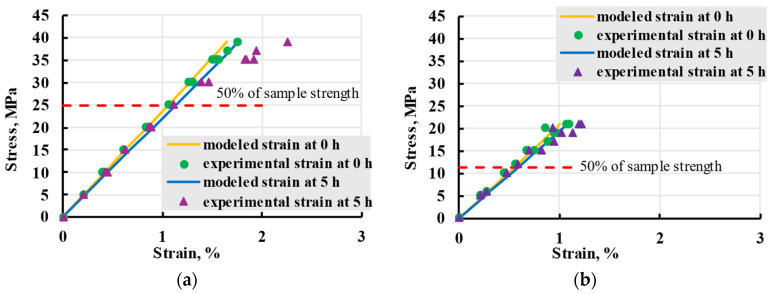
Creep isochrones of viscoelastic experiments for X (**a**) and Y (**b**) samples at *t* = 0 (elastic strain, green dots, yellow lines) and 5 h (blue triangles, blue lines).

The creep isochrones present data for all tested stress ranges (Figure 25). Modelled lines are close to experimental, up until roughly 50% of maximal strength when the experimental strain becomes larger.

**Figure 26:**
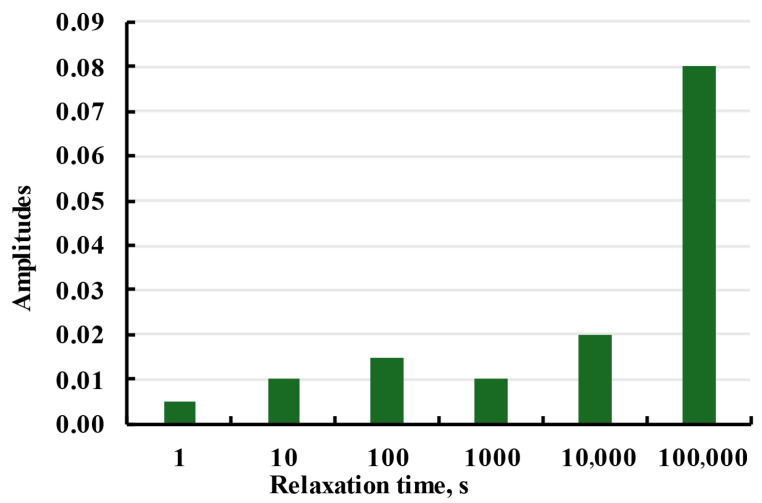
Relaxation spectrum used for the Prony series model.

All samples were modelled with the same relaxation spectrum presented in Figure 26. Parameters for relaxation spectrum were fitted using aim function as average absolute deviation of calculated creep strain from experimental data for all set of experiments. In this case, four set of experiments were X samples linear, X samples nonlinear, Y samples linear, and Y samples nonlinear. Because the same spectrum adequately fits the samples printed in both directions, it can be assumed that structural anisotropy does not influence the viscoelastic properties of the material, and the anisotropy could be taken into account with the sample elasticity.

#### 4.3.2. Nonlinear Model

**Figure 27:**
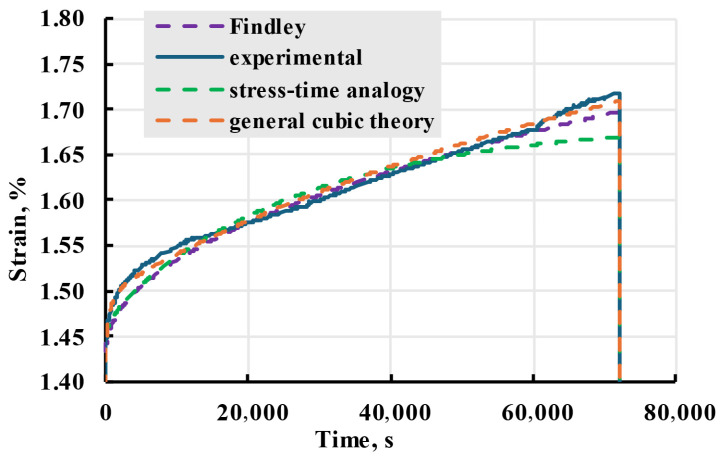
Comparison of different nonlinear viscoelastic models given in the legend with experimental data. Experimental results for X sample creep at 33 MPa.

The experimental creep strain data from X sample loaded at 33 MPa were used to evaluate nonlinear models (Figure 27). Among evaluated nonlinear models, Findley and main cubic theory were the most accurate for given data, while. With evaluation seen in Table 3 main cubic theory was chosen as model used for experimental data description.

**Figure 28:**
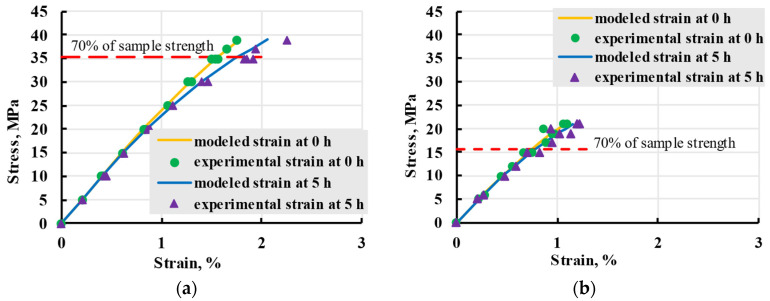
Isochrones of viscoelastic experiments for X (**a**) and Y (**b**) samples at 5 h with varied elastic modulus.

To accurately describe the viscoelastic properties of PETG samples in wide stress ranges, a nonlinear model based on general cubic theory was used. The same relaxation spectrum used for the linear model was used for nonlinear, with the addition of different elastic moduli based on changes observed during loading and unloading. Both sample groups were adequately described up 70% of sample strength when modelled with best-fitting elastic moduli, as seen in Figure 28.

**Figure 29:**
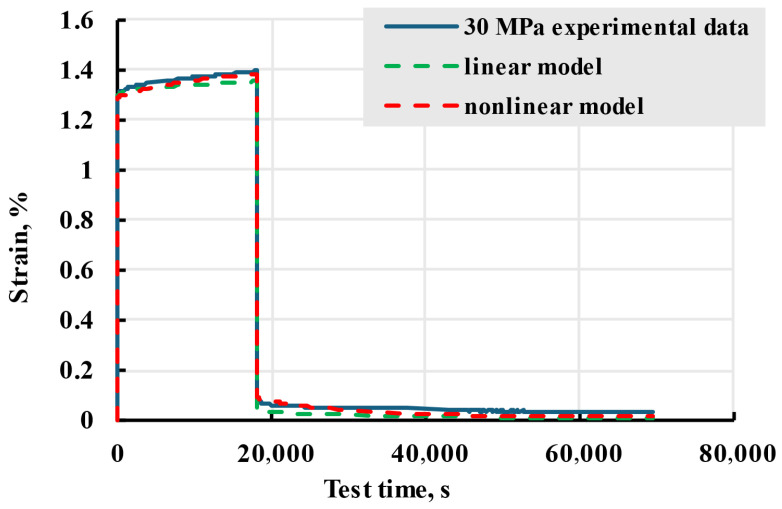
Experimental data of X sample creep experiment at stress of 30 MPa and modelled linear and nonlinear curves.

Comparing the results of linear and nonlinear models, as seen in Figure 29, an improvement in adherence to experimental data can be seen after switching from a linear to nonlinear model.

## 5. Conclusions

This study shows that printed PETG samples exhibit noticeable viscoelastic creep when subjected to constant stress. Samples under stress with less than 50% of their strength gained up to an additional 1% strain for X-direction samples and 0.6% for Y-direction samples in 5 h. The creep process of these samples can be described using a linear viscoelastic model based on the Boltzmann–Volterra theory and the Prony series. A nonlinear model based on general cubic theory was used to describe experimental data adequately for applied stress up to 70% of sample strength. The analytical modelling of multistep loading process was performed, which allowed for easier simulation of creep recovery process.

For the Prony series kernel with given loading times, it was determined that creep during loading stage can be neglected and all deformation during stress loading can be described with elastic response. The response itself changes with applied stress, its values being close for low and moderate stresses, but noticeably different for high stresses. This difference suggests accumulation of plastic deformations and material hardening, particularly after creep strain reaches 0.1% for X samples and 0.05% for Y samples.

The creep process of all tested samples was described using the same relaxation spectrum. The printing process introduces structural anisotropy in the specimens, but this could be considered with the specimen’s elastic properties while not affecting viscoelastic ones.

The reliability and repeatability of experiments can be improved by more precisely controlling room temperature and stress loading speed. Additional tests of samples printed with other alignments would improve the reliability of the result.

## Figures and Tables

**Table 1:** Characteristics of evaluated linear kernels. Green stands for the most fitting, yellow, for less fitting, and red for unsuitable parameters.

Kernel Model	Defined Elastic Modulus	Deformation at Infinite Time	Number of Parameters	Computing Complexity	Adherence to Exponent
Duffing	at *t* = *t*_0_	non-convergent	4	easy	low
Exponential	both	convergent	3	easy	high
Prony	at *t* = *t*_0_	convergent	3+	medium	high
Rzhanitsin	at *t* = inf	convergent	6	medium	medium
Rabotnov	at *t* = *t*_0_	non-convergent	5	hard	low
Abel	at *t* = *t*_0_	non-convergent	4	hard	low

**Table 3:** Characteristics of evaluated nonlinear models. Green stands for the most fitting, yellow, for less fitting, and red for unsuitable parameters.

Model	Relation to Linear Models	Nonlinear Elastic Response	Number of Nonlinear Parameters	Computational Complexity
Findley	None	Built-in	4	Low
Schapery	Medium	Can be modified	4	High
Stress-time analogy	High	Can be modified	2+	Low
Main cubic theory	High	Can be modified	2	Medium

**Table 4:** Constant stress applied in long- and short-term creep tests and their relation to the strength.

X Samples			Y Samples		
Stress, MPa	Stress Relative to Strength, %	Long- (L) or Short-Term (S)	Stress, MPa	Stress Relative to Strength, %	Long- (L) or Short-Term (S)
5	10	S	5	22	S
10	20	S	6	27	S
15	30	S	10	44	S
20	40	S	12	53	S
25	50	S	15	67	L, S
27	54	L	17	76	L, S
30	59	L, S	19	85	L, S
33	65	L	20	89	S
35	69	L, S	21	93	S
37	73	L, S			
39	77	S			
40	79	L			

## Data Availability

Aniskevich, A., & Stankevics, L., Wolfram Mathematica file “Viscoelastic Creep Models”, 2025, Zenodo, https://doi.org/10.5281/zenodo.15364577. The document shows different models investigated during this research and allows manual adjustment of model parameters and real-time response to changes. It also allows the exporting of data tables of modelled results for specific parameters of linear models.

## Supplementary Materials

The following supporting information can be downloaded at https://www.mdpi.com/article/10.3390/polym17152075/s1. File S1: CSV example file with Ultimaker Cura parameters used for printing Y samples. Figures S1 and S2: Two representative graphs of ambient temperature and moisture plotted against test time for Y sample loaded with 10 MPa.

## Appendix A

Because the whole experiment time is subdivided in (22), instead of one integral over all experiment time, a sum of integrals, each calculated from the respective step start until the step end, can be substituted in the following:

(A1) $$\int_{t_{1}}^{t}\sigma \left(\tau \right)e^{-\frac{t-\tau }{\eta_{i}}}d\tau =\int_{t_{1}}^{t_{2}}\sigma_{1}e^{-\frac{t-\tau }{\eta_{i}}}d\tau +\int_{t_{2}}^{t_{3}}\sigma_{2}e^{-\frac{t-\tau }{\eta_{i}}}d\tau +\ldots +\int_{t_{N-1}}^{t_{N}}\sigma_{N-1}e^{-\frac{t-\tau }{\eta_{i}}}d\tau +\int_{t_{N}}^{t}\sigma_{N}e^{-\frac{t-\tau }{\eta_{i}}}d\tau .$$

Because stress is a stepwise function, stress at each step remains constant and can be factored out from under the integration sign:

(A2) $$\begin{array}{l} \int_{t_{1}}^{t_{2}}\sigma_{1}e^{-\frac{t-\tau }{\eta_{i}}}d\tau +\int_{t_{2}}^{t_{3}}\sigma_{2}e^{-\frac{t-\tau }{\eta_{i}}}d\tau +\ldots +\int_{t_{N-1}}^{t_{N}}\sigma_{N-1}e^{-\frac{t-\tau }{\eta_{i}}}d\tau +\int_{t_{N}}^{t}\sigma_{N}e^{-\frac{t-\tau }{\eta_{i}}}d\tau =\\ =\sigma_{1}\int_{t_{1}}^{t_{2}}e^{-\frac{t-\tau }{\eta_{i}}}d\tau +\sigma_{2}\int_{t_{2}}^{t_{3}}e^{-\frac{t-\tau }{\eta_{i}}}d\tau +\ldots +\sigma_{N-1}\int_{t_{N-1}}^{t_{N}}e^{-\frac{t-\tau }{\eta_{i}}}d\tau +\sigma_{N}\int_{t_{N}}^{t}e^{-\frac{t-\tau }{\eta_{i}}}d\tau \end{array}.$$

The summands from 1 to *N* − 1 can be placed under the summation symbol with summation index *n*:

(A3) $$\begin{array}{l} \sigma_{1}\int_{t_{1}}^{t_{2}}e^{-\frac{t-\tau }{\eta_{i}}}d\tau +\sigma_{2}\int_{t_{2}}^{t_{3}}e^{-\frac{t-\tau }{\eta_{i}}}d\tau +\ldots +\sigma_{N-1}\int_{t_{N-1}}^{t_{N}}e^{-\frac{t-\tau }{\eta_{i}}}d\tau +\sigma_{N}\int_{t_{N}}^{t}e^{-\frac{t-\tau }{\eta_{i}}}d\tau =\\ =\sum_{n=1}^{N-1}\sigma_{n}\int_{t_{n}}^{t_{n+1}}e^{-\frac{t-\tau }{\eta_{i}}}d\tau +\sigma_{N}\int_{t_{N}}^{t}e^{-\frac{t-\tau }{\eta_{i}}}d\tau \end{array}.$$

Now, summands under the summation symbol can be calculated as an integral from *t*_n_ to *t*_n+1_:

(A4) $$\begin{array}{l} \sigma_{n}\int_{t_{n}}^{t_{n+1}}e^{-\frac{t-\tau }{\eta_{i}}}d\tau =\sigma_{n}\int_{t_{n}}^{t_{n+1}}e^{-\frac{t}{\eta_{i}}}e^{\frac{\tau }{\eta_{i}}}d\tau =\\ =\sigma_{n}e^{-\frac{t}{\eta_{i}}}\int_{t_{n}}^{t_{n+1}}e^{\frac{\tau }{\eta_{i}}}d\tau =\sigma_{n}e^{-\frac{t}{\eta_{i}}}\eta_{i}e^{\frac{\tau }{\eta_{i}}}{|}_{t_{n}}^{t_{n+1}}=\sigma_{n}e^{-\frac{t}{\eta_{i}}}\eta_{i}\left(e^{\frac{t_{n+1}}{\eta_{i}}}-e^{\frac{t_{n}}{\eta_{i}}}\right) \end{array}.$$

and the last summand can be calculated as

(A5) $$\begin{array}{l} \sigma_{N}\int_{t_{N}}^{t}e^{-\frac{t-\tau }{\eta_{i}}}d\tau =\sigma_{N}\int_{t_{N}}^{t}e^{-\frac{t}{\eta_{i}}}e^{\frac{\tau }{\eta_{i}}}d\tau =\\ =\sigma_{N}e^{-\frac{t}{\eta_{i}}}\eta_{i}e^{\frac{\tau }{\eta_{i}}}{|}_{t_{N}}^{t}=\sigma_{N}e^{-\frac{t}{\eta_{i}}}\eta_{i}\left(e^{\frac{t}{\eta_{i}}}-e^{\frac{t_{N}}{\eta_{i}}}\right)=\sigma_{N}\eta_{i}\left(1-e^{\frac{t_{N}-t}{\eta_{i}}}\right) \end{array}.$$

Substituting the results of the integration in Equation (12) results in

(A6) $$\begin{array}{l} \varepsilon \left(t\right)=\frac{\sigma_{N}}{E}+\overset{k}{\underset{i=1}{\sum }}\frac{A_{i}}{\eta_{i}E}\int_{0}^{t}\sigma \left(\tau \right)e^{-\frac{t-\tau }{\eta_{i}}}d\tau =\\ =\frac{\sigma_{N}}{E}+\overset{k}{\underset{i=1}{\sum }}\frac{A_{i}}{\eta_{i}E}\left[\overset{N-1}{\underset{n=1}{\sum }}\sigma_{n}e^{-\frac{t}{\eta_{i}}}\eta_{i}\left(e^{\frac{t_{n+1}}{\eta_{i}}}-e^{\frac{t_{n}}{\eta_{i}}}\right)+\sigma_{N}\eta_{i}\left(1-e^{\frac{t_{N}-t}{\eta_{i}}}\right)\right] \end{array}.$$

Finally,

(A7) $$\varepsilon \left(t\right)=\frac{\sigma_{N}}{E}+\overset{k}{\underset{i=1}{\sum }}\frac{A_{i}}{E}\left[\overset{N-1}{\underset{n=1}{\sum }}\sigma_{n}e^{-\frac{t}{\eta_{i}}}\left(e^{\frac{t_{n+1}}{\eta_{i}}}-e^{\frac{t_{n}}{\eta_{i}}}\right)+\sigma_{N}\left(1-e^{\frac{t_{N}-t}{\eta_{i}}}\right)\right]$$
