# Supplementary figures and images for: Viscoelastic Creep of 3D-Printed Polyethylene Terephthalate Glycol Samples
Source: Polymers (Basel). 2025 Jul 29;17(15):2075. doi: 10.3390/polym17152075 (PMC12349189; doi:10.3390/polym17152075)

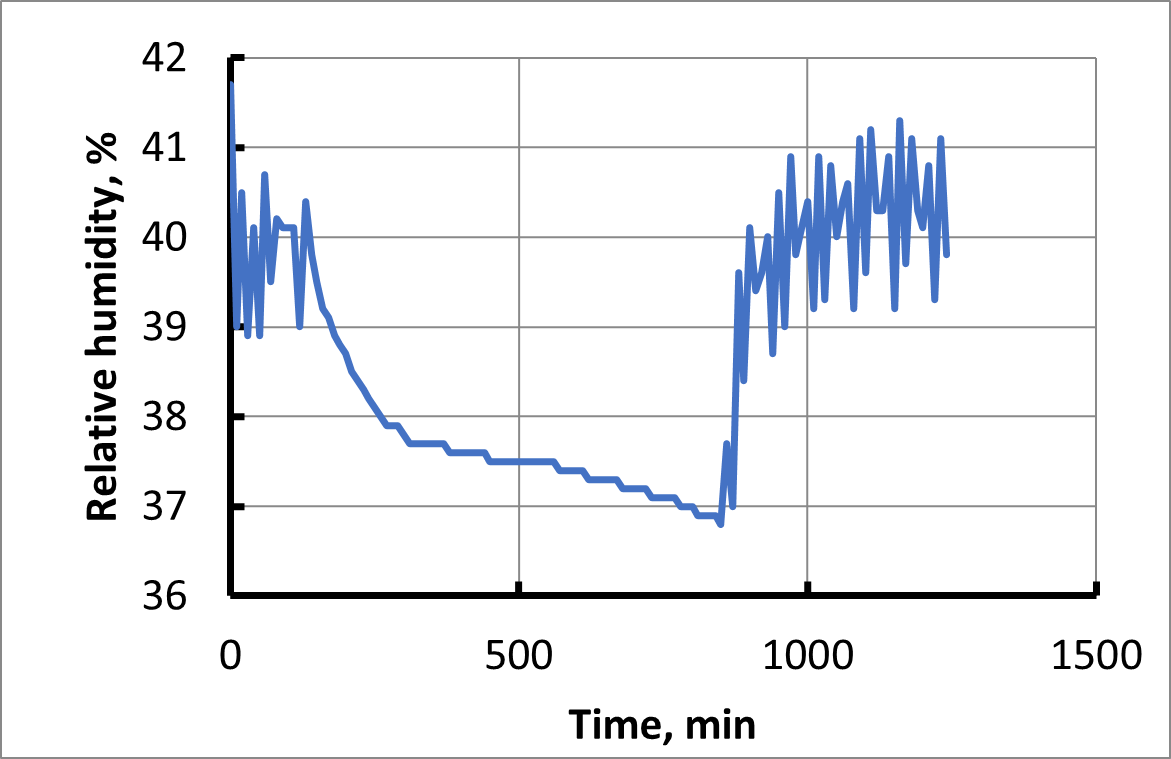

Supplement: Supplementary file 1 [file polymers-17-02075-s001.zip › polymers-3741254-supplementary/Figure S1. time_humidity.png]

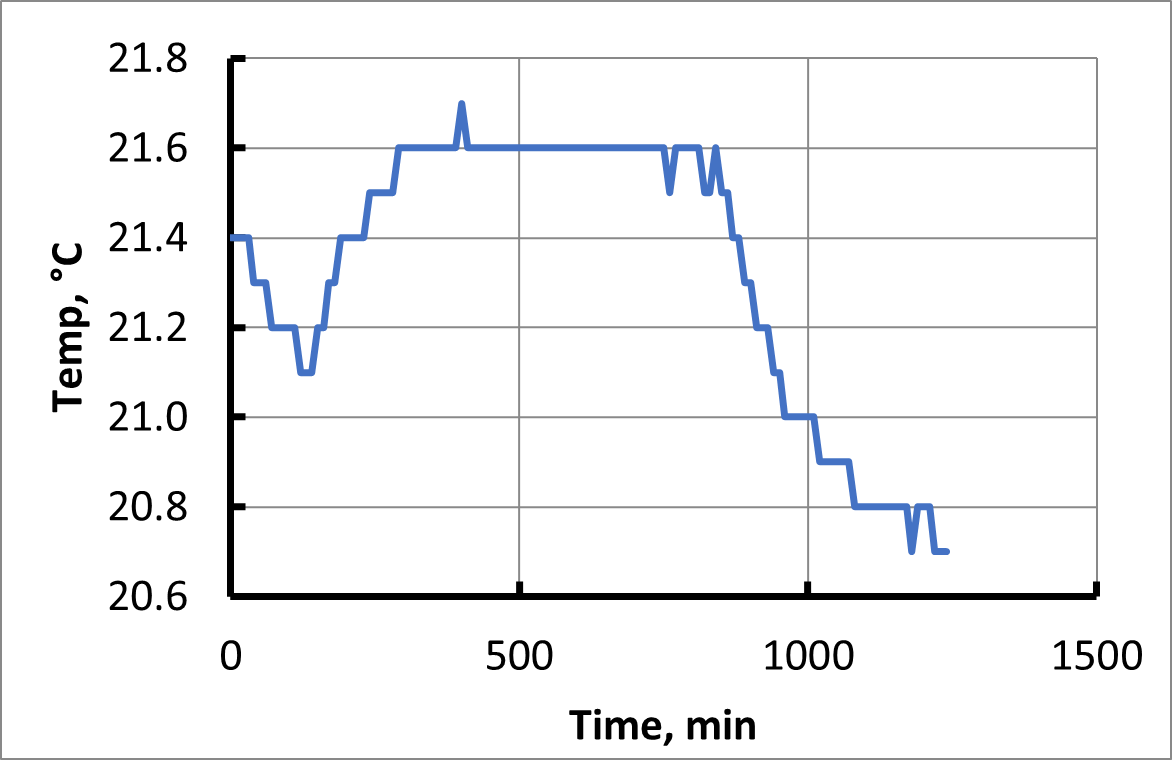

Supplement: Supplementary file 1 [file polymers-17-02075-s001.zip › polymers-3741254-supplementary/Figure S2. time_temperature.png]
